# Comparison of the Clinical Outcomes between Echelette Extended Range of Vision and Diffractive Bifocal Intraocular Lenses

**DOI:** 10.1155/2019/5815040

**Published:** 2019-09-23

**Authors:** Xin Liu, Xiaohui Song, Wei Wang, Yanan Zhu, Danni Lyu, Xingchao Shentu, Peiqing Chen, Yibo Yu, Ke Yao

**Affiliations:** Eye Center, The Second Affiliated Hospital of College of Medicine, Zhejiang University, Hangzhou, Zhejiang 310000, China

## Abstract

**Purpose:**

To compare the clinical outcomes of echelette extended range of vision (ERV) and diffractive bifocal intraocular lenses (IOLs).

**Methods:**

This is a prospective, consecutive, nonrandomized clinical trial. Seventy-three eligible patients (109 eyes) received the implantation of echelette ERV IOL (Tecnis Symfony ZXR00) or diffractive bifocal IOL (Tecnis ZMB00). 1 week, 1 month, and 3 months after surgery, visual acuities at different distances were examined. At 3 months, defocus curves, contrast sensitivities (CSs) with and without glare, optic path difference (OPD) scans, and questionnaires were evaluated. Regression analyses were applied to discover influence factors on postoperative vision.

**Results:**

ZXR00 showed better distance (*P* < 0.05) and intermediate (*P* < 0.001) visual acuities, while ZMB00 was better at distance-corrected near visual acuity (*P* < 0.001). Multivariate analyses indicated that worse intermediate (*P* < 0.001) and near vision (*P*=0.013) of ZMB00 might occur in patients with longer axial length. ZXR00 demonstrated smoother defocus curve and higher CSs. Superior modulation transfer function (MTF) and higher Strehl ratio (*P* < 0.05) were shown in ZXR00. In questionnaire evaluation, ZXR00 received better outcomes in self-reported vision, Visual Function-14 (VF-14) questionnaire, Quality of Vision (QoV) questionnaire, satisfaction, and recommendation grades. Spectacle dependence did not differ between ZXR00 and ZMB00 statistically.

**Conclusion:**

ZXR00 proved to be remarkable in distance and intermediate vision, defocus curve smoothness, CSs, and visual comfort, while ZMB00 achieved better near vision. ZXR00 may attain better near vision if postoperative SE remains slightly negative. Patients with relatively longer axial length might receive less favorable intermediate and near vision after ZMB00 implantation. This trial is registered with ChiCTR-ONC-17011119.

## 1. Introduction

Intraocular lens (IOL) implantation has become a common practice for the increasingly large population of cataract patients; however, it compromised ocular accommodating ability, leading to postoperative presbyopia and a high spectacle dependence rate up to 80% [1]. Multiple solutions, like the monovision of the 1950s, the bifocal IOLs of the 1980s, and the accommodating IOLs, trifocal IOLs, and extended range of vision (ERV) IOLs of the 21st century, were developed to tackle the problem.

Compared to monofocal IOLs, multifocal intraocular lenses (MIOLs) like bifocal and trifocal ones are able to provide clear images at each focus and alleviate the problem of presbyopia. They are mostly designed on the principles of diffraction and refraction. However, the modification of the light path by MIOLs has created new challenges such as dysphotopsia, decreased contrast sensitivity (CS), and compromised night vision [2].

ERV IOLs, on the other hand, were not approved by the U.S. Food and Drug Administration until 2016. Instead of adding certain focus, ERV IOLs extended the depth of focus. The effects of ERV IOLs were achieved based on the principles of echelette diffractive ring (Tecnis Symfony ZXR00), spherical aberration induction (SIFI MiniWell), or pinhole effect (Acu-Focus IC-8) [3]. Unlike MIOLs, ERV IOLs tend to retain CS to the similar level of monofocal IOLs [4].

Clinical trials have demonstrated the presbyopia-correcting effect in bifocal IOL [5] and ERV IOL [6], respectively, but direct comparison between diffractive echelette ERV IOL and diffractive bifocal IOL, which would be helpful to highlight the design of echelette apart from other confounding factors, remains to be rare. Although Black [7] and de Medeiros et al. [8] reported visual outcomes after blended implantation of diffractive echelette ERV IOL and diffractive bifocal IOL, thorough evaluations including visual acuity, defocus curve, CS, modulation transfer function (MTF), Strehl ratio, and subjective evaluation that stress the difference between these 2 IOLs were necessary to provide optimal IOL-selection strategies.

This study chose Tecnis Symfony ZXR00, the most widely used diffractive echelette ERV IOL, and Tecnis ZMB00, a diffractive bifocal IOL commonly applied in our center as well as an analogous to ZXR00, to analyze their differences on clinical performance.

## 2. Materials and Methods

### 2.1. Patient Enrolment

This prospective, consecutive, nonrandomized clinical trial was conducted at the Eye Center, the Second Affiliated Hospital of College of Medicine, Zhejiang University, Hangzhou, Zhejiang, China, from August 2016 to March 2018.

Patients diagnosed with cataract and interested in presbyopia correction were informed about the study. Thorough examinations were performed to select eligible participants. Inclusion criteria were as follows: (a) age from 50 to 80 years old; (b) cataract nuclear density Emery Grade I to III; (c) axial length from 21.0 to 26.0 mm; (d) angle kappa no more than 0.5 mm; (e) corneal astigmatism within 4.0 mm zone no more than 1.5 diopters (D); and (f) corneal endothelial cell count (measured by Noncon ROBO Pachy SP-9000, Konan Medical, Inc., Tokyo, Japan) no less than 1500/mm^2^. Patients were excluded if they had any of the following: (a) ocular comorbidities that would influence postoperative visual acuity; (b) previous ocular surgeries; (c) traumatic cataract; (d) unstable posterior capsule or loose zonular fibers; and (e) severe systemic diseases that would disable the cooperation with postoperative examinations. Seventy-three eligible patients were consecutively enrolled. Patients who had definite requirement on intermediate vision (such as TV watching, board games, and household duties) were implanted with ZXR00, while those who required definite near vision (such as reading, writing, and knitting) were implanted with ZMB00. The investigators of postoperation examinations and patients themselves were masked to the type of IOLs implanted.

### 2.2. Intraocular Lenses

Tecnis Symfony ZXR00 (Johnson & Johnson Vision, Santa Ana, California, USA) is a hydrophobic UV-filtering C-loop IOL. With an overall diameter of 13.0 mm and an optic diameter of 6.0 mm [9], the acrylic acid IOL is a biconvex. Its anterior surface is designed to provide a negative spherical aberration of 0.27 *μ*m. Its posterior surface is composed of an achromatic design and an echelette, a special type of diffraction grating [10], to extend the range of vision. The refractive area within the 9 rings of diffractive zone has a diameter of 1.7 mm. Its light utilization ratio is 92%.

Tecnis ZMB00 (Johnson & Johnson Vision, Santa Ana, California, USA) shared a similar design as ZXR00 except that its posterior surface is composed of 22 concentric diffractive rings, providing a near addition of +4.0 D (+3.0 D on spectacle plane) [11]. The refractive area within the diffractive zone has a diameter of 1.0 mm, and the light efficiency is about 82%, with a 1 : 1 distribution between two foci.

### 2.3. Surgical Procedure

IOL power was chosen to target within 0.5 D deviation from emmetropia. All surgeries were performed under topical anesthesia by 4 senior surgeons, each with experience of more than 10,000 cases of cataract surgeries. The IOLs were implanted through a 2.0 mm limbal corneal incision. Standard phacoemulsification or femtosecond laser-assisted technique was carried out depending on the preference of the participants. Postoperative topical therapy included dexamethasone-tobramycin for 2 weeks and pranoprofen for 1 month.

### 2.4. Patient Examinations

Under consistent environmental lighting condition, patients were examined at 5 m, 80 cm, and 40 cm for monocular uncorrected (UCDVA) and corrected (CDVA) distance visual acuities, monocular uncorrected (UCIVA) and corrected (CIVA) intermediate visual acuities, monocular uncorrected (UCNVA) and corrected (CNVA) near visual acuities, as well as monocular distance-corrected intermediate (DCIVA) and near (DCNVA) visual acuities. In addition, monocular defocus curves from +2.5 D to −4.0 D based on best distance-corrected status were also detected. CS with and without glare under mesopic condition was measured by Glare Tester CGT-1000 (Takagi Seiko Co., Ltd., Japan) based on best near-corrected status. 0.5% tropicamide was used for pupil dilation in order to complete optic path difference (OPD) scan (OPD-Scan II, Nidek Co., Ltd., Japan) within 3.0 mm and 5.0 mm pupil. Furthermore, an assessor-directed questionnaire that included Visual Function-14 (VF-14) questionnaire [12], Quality of Vision (QoV) questionnaire [13], day vision score, night vision score, spectacle dependence, satisfaction grade, and recommendation grade was completed at the last visit for every operation. In particular, the final score of VF-14 was calculated as the total scores divided by the number of questions effectively answered (thus excluding “not applicable” responses), multiplied by 25, and then deducted from 100 [14].

### 2.5. Statistical Analyses

Statistical analyses were performed using SPSS 19.0 for Windows (SPSS, Inc., Chicago, Illinois, USA). The normality of data was evaluated using the Kolmogorov–Smirnov test. Comparisons between 2 groups were made by *t*-tests or Wilcoxon–Mann–Whitney *U*-tests, depending on data normality and homogeneity of variance. Repeated measures one-way ANOVAs were applied for comparison across time, while post hoc Bonferroni tests were applied when needed. For categorical data, Chi-square tests were applied. STATA 13 (StataCorp LLC, College Station, Texas, USA) was used for multivariate analyses with linear regressions. *P* values less than 0.05 were considered statistically significant. All tests were analyzed in two-tailed style.

## 3. Results

A total of 73 patients (109 eyes) attended to at least 1 follow-up visit. Missing data were due to personal inconvenience, refusal to mydriasis for OPD scan, or temporary device failure. A total of 38 patients (56 eyes) were implanted with ZXR00, while 35 patients (53 eyes) were implanted with ZMB00. No significant difference was found between the 2 groups regarding preoperative characteristics (Table 1). No intraoperative complication occurred.

### 3.1. Visual Acuities

39 eyes implanted with ZXR00 and 28 eyes implanted with ZMB00 completed all 3 follow-up visits, where repeated measurements of uncorrected visual acuities and spherical equivalent (SE) showed no significant change within either group, except that better UCNVA was gained in ZXR00 after 1 month (*P*=0.008) (Table 2).

Table 3 shows ZXR00 achieved better outcomes in UCDVA (*P*=0.012) and UCIVA (*P* < 0.001), as well as in CDVA (*P*=0.008) and DCIVA (*P* < 0.001), while ZMB00 proved to be excellent in DCNVA (*P*=0.001); no significant differences were discovered between the 2 groups regarding UCNVA, CIVA, and CNVA. Table 3 also shows that patients implanted with ZXR00 required less spectacle correction of SE to gain the best intermediate vision (*P*=0.036), but required more to gain the best near vision (*P* < 0.001) than patients implanted with ZMB00. Postoperative SE between the 2 groups differed (*P*=0.025), with the ZXR00 group being relatively more myopic.

Multivariate analysis (Table 4) after adjustment of age, gender, keratometry, and anterior chamber depth suggested the negative effect of longer axial length on DCIVA in ZMB00 (*P* < 0.001, 95% CI 0.067∼0.209). In the analysis of DCIVA in ZXR00, no significant correlation was detected among the observed factors.

The relation between longer axial length and worse vision was also revealed in the DCNVA of ZMB00 (*P*=0.013, 95% CI 0.030∼0.238) (Table 5). In the analysis of DCNVA in ZXR00, only age stood out as potential relative factor in the multivariate model, indicating ZXR00 implanted eyes achieved better DCNVA in older patients (*P*=0.018, 95% CI −0.015∼−0.002).

### 3.2. Defocus Curve

**Figure 1:**
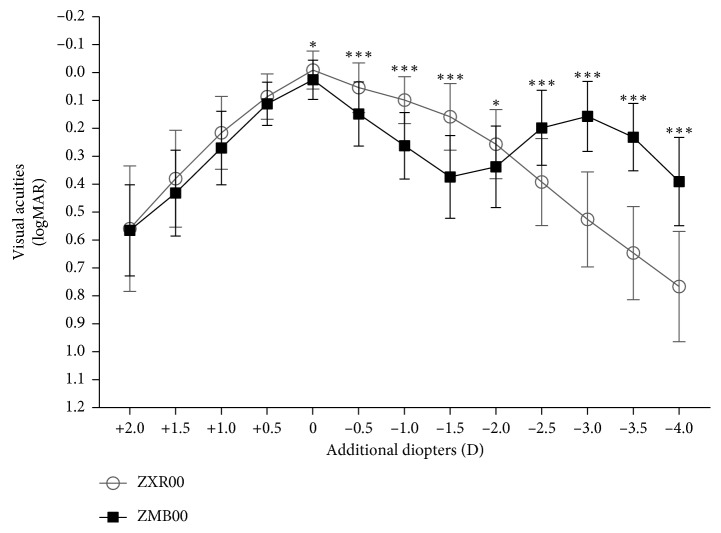
Defocus curves 3 months after IOL implantation (D = diopter; ^*∗*^ = *P* < 0.05; ^*∗∗*^ = *P* < 0.01; ^*∗∗∗*^ = *P* < 0.001).

Defocus curve was tested with every increment of 0.5 D 3 months after surgery (Figure 1). In contrast to ZMB00, ZXR00 advanced in defocus curve from 0 D to −2.0 D but lagged from −2.5 D to −4.0 D. Overall, the curve of ZXR00 was smooth, while ZMB00 peaked at 0 D and −3.0 D.

### 3.3. Contrast Sensitivity

**Figure 2:**
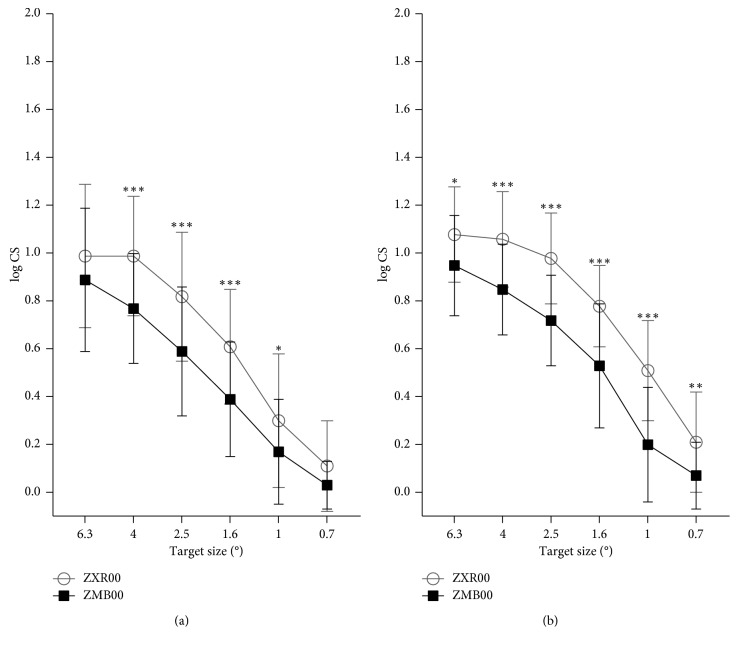
Contrast sensitivities with (a) and without (b) glare under mesopic condition 3 months after IOL implantation (CS = contrast sensitivity; ° = degree of angle; ^*∗*^ = *P* < 0.05; ^*∗∗*^ = *P* < 0.01; ^*∗∗∗*^ = *P* < 0.001).

Either with glare (Figure 2(a)) or without glare (Figure 2(b)), ZXR00 achieved higher CS at nearly all ranges of spatial frequency, especially at medium spatial frequency (target sizes of 2.5 and 1.6 degree).

### 3.4. Optic Path Difference Scan

**Figure 3:**
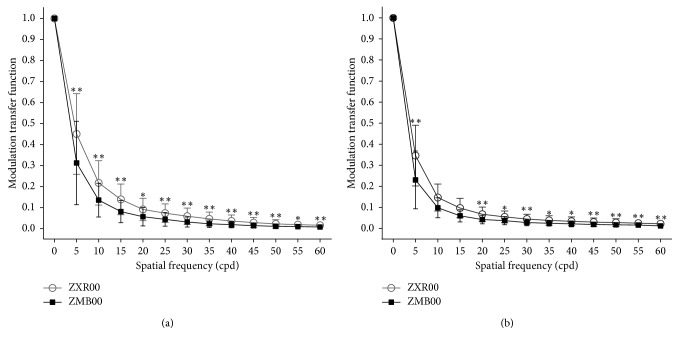
Modulation transfer function for 3.0 mm (a) and 5.0 mm (b) pupil 3 months after IOL implantation (cpd = cycle per degree; ^*∗*^ = *P* < 0.05; ^*∗∗*^ = *P* < 0.01; ^*∗∗∗*^ = *P* < 0.001).

3 months after operation, 34 of the ZXR00 implanted eyes and 26 of the ZMB00 implanted eyes received effective OPD scans. ZXR00 exceeded in modulation transfer function (MTF) values at overall spatial frequency for 3.0 mm (Figure 3(a)) and 5.0 mm (Figure 3(b)) pupil. Strehl ratio was also higher in ZXR00 than in ZMB00 for either 3.0 mm (0.06 ± 0.06 vs. 0.03 ± 0.03, *P*=0.021) or 5.0 mm (0.02 ± 0.01 vs. 0.01 ± 0.01, *P*=0.005) pupil.

### 3.5. Questionnaire Evaluation

A total of 98 eyes completed subjective evaluations 3 months after operation.  Table 6 shows better outcomes in the ZXR00 group, including greater VF-14 score, lower QoV score, higher self-reported vision score (day and night), higher satisfaction grade, and higher recommendation grade (all *P* < 0.05). Spectacle dependence showed no statistical difference between the 2 groups (*P*=0.426). Only 1 female patient, aged 77 years old, who had her right eye implanted with ZMB00, demanded IOL explantation because of severe glare.

## 4. Discussion

This prospective study compared clinical outcomes of an echelette ERV IOL and a diffractive bifocal IOL with similar structures but different optic principles.

As the overall visual performance stabilized after 1 month, clinical outcomes at 3 months after operation were presented. ZXR00 showed better UCDVA and UCIVA, while the difference in UCNVA was not significant. As the postoperative SE differed between the 2 groups, spectacle-corrected vision performance should be taken into consideration. In this way, ZXR00 still advanced in CDVA and DCIVA, but fell behind in DCNVA (0.38 ± 0.17 logMAR). Consistently, patients in the ZXR00 group required fewer positive diopter additions of spectacle to gain the best intermediate vision, but more to gain the best near vision.

The superiority of UCDVA and CDVA in ZXR00 over ZMB00 could be explained by its achromatic design [15]; a clinical study showed better distance acuity in ZXR00 compared not only to MIOLs, but also to monofocal IOLs [16]. Better UCIVA and DCIVA, on the other hand, reflect the structure of diffractive echelette in ZXR00 to extend the depth of focus. The “extended range” of ZXR00 failed to cover the near range, resulting in a poorer DCNVA. Our result of monocular DCNVA at 40 cm (0.38 ± 0.17 logMAR) in ZXR00 is consistent with the studies of Pedrotti (0.33 ± 0.10 logMAR) [4], Hogarty (0.31 ± 0.10 logMAR) [17], and Pilger (0.33 ± 0.12 logMAR) [18]. Nevertheless, our study found that a little negative postoperative SE could compensate for this disadvantage by improving the UCNVA, a strategy similarly indicated by the study of Cocherner et al. [19, 20], who found that a micro-monovision of −0.5 D myopia in 1 eye led to better visual outcome for ZXR00 implantation. For ZMB00, it should be cautiously implanted in people with longer axial length, for it is correlated with worse intermediate and near vision based on our analyses.

In consistent with the visual acuities of different distances, the smooth defocus curve of ZXR00 excelled from 0 D to −2.0 D but fell behind ZMB00 from −2.5 D to −4.0 D. As for CS, ZXR00 overwhelmingly exceeded ZMB00. The target size of CS showed spatial frequency range from 6 to 12 cycles per degree (cpd) [21]. Target sizes of 6.3° and 4° represent low spatial frequency related to the magnocellular pathway, which is involved in recognizing moving objects [22]. Target sizes of 1° and 0.7°, on the contrary, represent high spatial frequency related to the parvocellular pathway, which is involved in recognizing object details [22]. Popularization of MIOLs has been challenged by compromised CS, especially under glare conditions [23], which could endanger night drivers. However, previous in vitro [24] and clinical [16] researchers stated that ZXR00 rivaled monofocal IOLs in CS. The consistent advantage of better CS in ZXR00 over ZMB00 here may be attributed to its fewer diffractive rings and achromatic designs [25].

OPD scans showed that ZXR00 implantation resulted in higher MTF values and Strehl ratio, which were consistent with its excellent distance visual acuity and CS.

The questionnaire analyses uncovered that although near vision was compromised in ZXR00, its spectacle dependence was no more than that of ZMB00. Similar results were seen in previous nonrandomized [16] and randomized [26] studies, where, despite the poorer near vision of ZXR00, the spectacle dependency rate did not differ significantly from a +3.0 D bifocal IOL, or from a trifocal IOL that had near addition powers of +2.17 D and +3.25 D. This could be attributed to the smooth defocus curve of ZXR00 allowing patients the convenience of slightly adjusting reading distance for better vision. ZXR00 even reported higher VF-14 score, self-reported vision score, satisfaction grade, and recommendation grade.

One study showed that glare, one of the most commonly seen photic phenomena of presbyopia-correcting IOLs [26–29], appeared at comparable frequency between ZXR00 and apodized diffractive-refractive bifocal IOLs [16]. But our study revealed better visual quality of ZXR00 by achieving lower QoV score than ZMB00, especially in the bothersome subscale.

Limitations existed in our study, though, as it was not a randomized clinical trial with a 100% follow-up rate and unanimous bilateral IOL implantation due to patients' compliance. In addition, the possible correlation between axial length and visual outcomes in ZMB00 needs further exploration.

## 5. Conclusions

In conclusion, our study provides certain clinical advice in choosing presbyopia-correcting IOLs. ZXR00 is outstanding in distance and intermediate visual acuities, smooth defocus curve, high CS, and fair visual comfort. ZXR00 may attain better near vision if postoperative SE remains slightly negative. ZMB00 is better in near vision, but patients like night drivers should be cautious because of its lower CS and more visual disturbances. Patients with relatively longer axial length should also be informed about less favorable vision before implanted with ZMB00.

## Figures and Tables

**Table 1:** Participant characteristics.

Parameter	ZXR00	ZMB00	*P* value
Patients/eyes (*n*)	38/56	35/53	
Implantation type			0.840
Monocular (*n*)	20 (35.7%)	17 (32.1%)	
Binocular (*n*)	36 (64.3%)	36 (67.9%)	
Age (years), mean ± SD	68.77 ± 8.22	66.87 ± 6.53	0.186
Gender			0.825
Male (*n*)	13 (23.2%)	14 (26.4%)	
Female (*n*)	43 (76.8%)	39 (73.6%)	
UCDVA (logMAR), mean ± SD	0.58 ± 0.38	0.70 ± 0.45	0.145
Keratometry (D), mean ± SD	43.78 ± 1.51	43.90 ± 1.43	0.677
Axial length (mm), mean ± SD	23.65 ± 0.70	23.73 ± 0.94	0.627
Anterior chamber depth (mm), mean ± SD	2.81 ± 0.46	2.76 ± 0.45	0.604
IOL power (D), mean ± SD	21.12 ± 1.49	20.83 ± 2.13	0.417
Angle kappa (mm), mean ± SD	0.20 ± 0.11	0.24 ± 0.14	0.176
Corneal astigmatism (D), mean ± SD	0.58 ± 0.22	0.53 ± 0.27	0.314
Corneal endothelial cell count (mm^2^), mean ± SD	2532.0 ± 260.4	2596.21 ± 234.9	0.180
Nuclear hardness			0.337
Emery Grade I (*n*)	20 (35.7%)	21 (39.6%)	
Emery Grade II (*n*)	30 (53.6%)	22 (41.5%)	
Emery Grade III (*n*)	6 (10.7%)	10 (18.9%)	
Surgical technique			0.099
Standard (*n*)	22 (39.3%)	13 (24.5%)	
Femtosecond-assisted (*n*)	34 (60.7%)	40 (75.5%)	

*n*  number of eyes; UCDVA = uncorrected distance visual acuity; logMAR = logarithm of the minimum angle of resolution; mm = millimeter; D = diopter; IOL = intraocular lens; SD = standard deviation.

**Table 2:** Repeated measurements of visual acuities after IOL implantation.

Parameter	Postoperative visit			*P* value
	1 week	1 month	3 months	
UCDVA (logMAR), mean ± SD
ZXR00 (eyes = 39)	0.10 ± 0.14	0.11 ± 0.15	0.09 ± 0.13	0.606
ZMB00 (eyes = 28)	0.22 ± 0.21	0.19 ± 0.16	0.19 ± 0.17	0.415
UCIVA (logMAR), mean ± SD
ZXR00 (eyes = 39)	0.19 ± 0.16	0.18 ± 0.17	0.14 ± 0.12	0.161
ZMB00 (eyes = 28)	0.38 ± 0.24	0.35 ± 0.15	0.30 ± 0.15	0.112
UCNVA (logMAR), mean ± SD
ZXR00 (eyes = 39)	0.44 ± 0.19	0.35 ± 0.20^†^	0.34 ± 0.20^†^	0.008
ZMB00 (eyes = 28)	0.32 ± 0.18	0.28 ± 0.19	0.25 ± 0.18	0.126
SE (D), mean ± SD
ZXR00 (eyes = 39)	−0.19 ± 0.49	−0.21 ± 0.60	−0.19 ± 0.64	0.964
ZMB00 (eyes = 28)	0.23 ± 0.69	0.22 ± 0.71	0.12 ± 0.79	0.088

^†^ *P* < 0.05 compared to the visual acuity of 1 week after operation. UCDVA = uncorrected distance visual acuity; UCIVA = uncorrected intermediate visual acuity; UCNVA = uncorrected near visual acuity; SE = spherical equivalent; D = diopter; SD = standard deviation.

**Table 3:** Visual acuities and refractive outcomes 3 months after IOL implantation.

	ZXR00 (eyes = 45)	ZMB00 (eyes = 34)	*P* value
UCDVA (logMAR), mean ± SD	0.10 ± 0.13	0.19 ± 0.19	0.012
UCIVA (logMAR), mean ± SD	0.15 ± 0.13	0.29 ± 0.17	<0.001
UCNVA (logMAR), mean ± SD	0.35 ± 0.19	0.26 ± 0.21	0.057
CDVA (logMAR), mean ± SD	−0.01 ± 0.07	0.03 ± 0.08	0.008
CIVA (logMAR), mean ± SD	0.03 ± 0.11	0.08 ± 0.15	0.134
CNVA (logMAR), mean ± SD	0.10 ± 0.20	0.10 ± 0.18	0.719
DCIVA (logMAR), mean ± SD	0.12 ± 0.13	0.32 ± 0.19	<0.001
DCNVA (logMAR), mean ± SD	0.38 ± 0.17	0.22 ± 0.24	0.001
Int SE add (D), mean ± SD	0.72 ± 0.52	0.94 ± 0.38	0.036
Near SE add (D), mean ± SD	1.84 ± 0.62	0.45 ± 1.14	<0.001
SE (D), mean ± SD	−0.22 ± 0.61	0.12 ± 0.73	0.025

UCDVA = uncorrected distance visual acuity; UCIVA = uncorrected intermediate visual acuity; UCNVA = uncorrected near visual acuity; CDVA = corrected distance visual acuity; CIVA = corrected intermediate visual acuity; CNVA = corrected near visual acuity; DCIVA = distance-corrected intermediate visual acuity; DCNVA = distance-corrected near visual acuity; SE = spherical equivalent; int SE add = addition of diopters to spectacle from the best corrected distance vision to achieve the best corrected intermediate vision; near SE add = addition of diopters to spectacle from the best corrected distance vision to achieve the best corrected near vision; D = diopter; SD = standard deviation.

**Table 4:** Multivariate analysis on the DCIVA (logMAR) 3 months after IOL implantation.

IOL	Correlation indicators	Variables				
		Age (year)	Gender^†^	Keratometry (D)	Axial length (mm)	Anterior chamber depth (mm)
ZXR00 (eyes = 45)	Coefficient	−0.001	0.024	0.018	0.039	−0.012
	*P* value	0.810	0.730	0.326	0.334	0.811
	LCI	−0.007	−0.115	−0.019	−0.042	−0.116
	UCI	0.005	0.163	0.055	0.120	0.092
ZMB00 (eyes = 34)	Coefficient	0.007	0.147	0.024	0.138	−0.002
	*P* value	0.154	0.053	0.401	<0.001	0.978
	LCI	−0.003	−0.002	−0.034	0.067	−0.153
	UCI	0.016	0.296	0.082	0.209	0.149

^†^For gender, 0 indicates male while 1 indicates female. DCIVA = distance-corrected intermediate visual acuity; IOL = intraocular lens; D = diopter; LCI = lower bound of 95% confidence interval; UCI = upper bound of 95% confidence interval.

**Table 5:** Multivariate analysis on the DCNVA (logMAR) 3 months after IOL implantation.

IOL	Correlation indicators	Variables				
		Age (year)	Gender^†^	Keratometry (D)	Axial length (mm)	Anterior chamber depth (mm)
ZXR00 (eyes = 45)	Coefficient	−0.008	0.015	0.008	0.003	−0.043
	*P* value	0.018	0.852	0.705	0.956	0.485
	LCI	−0.015	−0.148	−0.035	−0.092	−0.165
	UCI	−0.002	0.178	0.052	0.098	0.080
ZMB00 (eyes = 34)	Coefficient	0.009	0.116	0.009	0.134	−0.144
	*P* value	0.187	0.286	0.822	0.013	0.192
	LCI	−0.004	−0.102	−0.075	0.030	−0.364
	UCI	0.022	0.333	0.094	0.238	0.077

^†^For gender, 0 indicates male while 1 indicates female. DCNVA = distance-corrected near visual acuity; D = diopter; LCI = lower bound of 95% confidence interval; UCI = upper bound of 95% confidence interval.

**Table 6:** Subjective evaluation by questionnaire 3 months after IOL implantation.

Questionnaire	ZXR00 (eyes = 54)	ZMB00 (eyes = 44)	*P* value
VF-14 score, mean ± SD	90.54 ± 12.63	85.54 ± 13.34	0.021
QoV score, mean ± SD	5.06 ± 6.15	8.54 ± 8.35	0.022
Frequency score	2.20 ± 2.43	3.39 ± 2.99	0.042
Severity score	1.65 ± 2.13	2.64 ± 2.69	0.023
Bothersome score	1.20 ± 1.74	2.52 ± 2.82	0.006
Self-reported vision			
Day score, mean ± SD	9.30 ± 1.24	8.39 ± 1.54	0.001
Night score, mean ± SD	8.74 ± 1.46	7.73 ± 1.88	0.004
Spectacle dependence			0.426
Independent (*n*)	32 (59.3%)	21 (47.7%)	
Occasionally (*n*)	18 (33.3%)	17 (38.7%)	
Often (*n*)	4 (7.4%)	6 (13.6%)	
Most of time (*n*)	0	0	
Always (*n*)	0	0	
Satisfaction grade			0.045
Very satisfied (*n*)	26 (48.1%)	11 (25.0%)	
Good (*n*)	19 (35.2%)	17 (38.6%)	
Partial improvement (*n*)	9 (16.7%)	12 (27.3%)	
Little improvement (*n*)	0	2 (4.5%)	
No improvement (*n*)	0	1 (2.3%)	
Worse (*n*)	0	1 (2.3%)	
Recommendation grade			0.045
Strong (*n*)	24 (44.4%)	11 (25.0%)	
Possible (*n*)	17 (31.5%)	13 (29.6%)	
Probable (*n*)	13 (24.1%)	18 (40.9%)	
Against (*n*)	0	2 (4.5%)	
No opinion (*n*)	0	0	

*n*  number of eyes; VF-14 = Visual Function-14; QoV = Quality of Vision; SD = standard deviation.

## Data Availability

The data used to support the findings of this study are available from the corresponding author upon request.
